# Cumulative impact of health deficits, social vulnerabilities, and protective factors on cognitive dynamics in late life: a multistate modeling approach

**DOI:** 10.1186/s13195-015-0120-7

**Published:** 2015-06-05

**Authors:** Joshua J Armstrong, Arnold Mitnitski, Melissa K Andrew, Lenore J Launer, Lon R White, Kenneth Rockwood

**Affiliations:** Geriatric Medicine Research, Faculty of Medicine, Dalhousie University, Halifax, NS Canada; Department of Medicine, Division of Geriatric Medicine, Dalhousie University, Halifax, NS Canada; Laboratory of Epidemiology, Demography, and Biometry, National Institute on Aging, Bethesda, MD USA; Pacific Health Research & Education Institute, Honolulu, HI USA

## Abstract

**Introduction:**

Many factors influence late-life cognitive changes, and evaluating their joint impact is challenging. Typical approaches focus on average decline and a small number of factors. We used multistate transition models and index variables to look at changes in cognition in relation to frailty (accumulation of health deficits), social vulnerability, and protective factors in the Honolulu-Asia Aging Study (HAAS).

**Methods:**

The HAAS is a prospective cohort study of 3,845 men of Japanese descent, aged 71 to 93 years at baseline. Cognitive function was measured using the Cognitive Abilities Screening Instrument (CASI). Baseline index variables were constructed of health deficits (frailty), social vulnerabilities, and protective factors. The chances of improvement/stability/decline in cognitive function and death were simultaneously estimated using multistate transition modeling for 3- and 6-year transitions from baseline.

**Results:**

On average, CASI scores declined by 5.3 points (standard deviation (SD) = 10.0) over 3 years and 9.5 points (SD = 13.9) over 6 years. After adjusting for education and age, baseline frailty was associated with an increased risk of cognitive decline at 3 years (β = 0.18, 95% confidence interval (CI), 0.08 to 0.29) and 6 years (β = 0.40, 95% CI, 0.27 to 0.54). The social vulnerability index was associated with 3-year changes (β = 0.16, 95% CI, 0.09 to 0.23) and 6-year changes (β = 0.14, 95% CI, 0.05 to 0.24) in CASI scores. The protective index was associated with reductions in cognitive decline over the two intervals (3-year: β = −0.16, 95% CI, −0.24 to −0.09; 6-year: β = −0.21, 95% CI, −0.31 to –0.11,).

**Conclusions:**

Research on cognition in late life needs to consider overall health, the accumulation of protective factors, and the dynamics of cognitive change. Index variables and multistate transition models can enhance understanding of the multifactorial nature of late-life changes in cognition.

**Electronic supplementary material:**

The online version of this article (doi:10.1186/s13195-015-0120-7) contains supplementary material, which is available to authorized users.

## Introduction

Although many population and longitudinal studies have identified a range of risk factors associated with cognitive decline, our ability to effectively treat cognitive impairment remains inadequate. One challenge in unraveling this problem is that, for any population, the cognition of individuals does not change uniformly. Cognitive decline occurs at different ages and at different rates of change, and some individuals do not decline at all [1-4]. Cognition in late life can also improve, as confirmed by examining rates of “reversion” from mild cognitive impairment back to normal cognitive states [5-11]. Even though people who improve are at greater risk for subsequent decline [11] some people remain in a cognitive normal state [11-13]. This variability in cognitive change appears to conform to a stochastic process in which change in cognition is not deterministic, but is conditional on the baseline status and fits known probability distributions [14-16].

To address the stochastic nature of transitions in cognition, our research group has developed a transition model based upon a parametric representation of a multistate Poisson model [14]. This approach allows for models that can account for change in any direction, and that simultaneously can incorporate the risk of death. Multistate transition models can also be modified to incorporate covariates into the analysis so that the effects of modifiers of interest can be evaluated; these have been applied to both transitions cognitive test scores [15] and changes in brain structure [16]. Here, our objective was to understand how cognition changes in late life in relation to frailty, social vulnerability, and the cumulative impact of multiple protective factors.

## Methods

### Study population

We used data from the Honolulu-Asia Aging Study (HAAS). The HAAS is a prospective cohort study of 3,845 Japanese-American men, free of dementia at baseline. It originated in the Honolulu-Heart Program, the participants of which were born between 1900 and 1919 and lived in Oahu, Hawaii, at baseline, as detailed elsewhere [17]. In 1991, the HAAS was established to focus on cognition and brain diseases. Follow-up examinations took place approximately every 3 years. Clinical assessments were conducted, as were structured interviews on participant characteristics. All dates of death for HAAS participants within the study period (1991 to 1997) were recorded by the study’s surveillance system.

### Cognitive assessments

The Cognitive Abilities Screening Instrument (CASI) [18], a 100-point scale designed for cross-cultural use, was administered at each study wave. It is a measure of global cognitive function that assesses attention, concentration, orientation, short-term memory, long-term memory, language abilities, visual construction, list-generating fluency, abstraction, and judgment. The CASI has been validated for use among Japanese and Western populations [19] and within the items subsets can be used to form the Hasegawa Dementia Screening Scale, the Folstein Mini-Mental State Examination, and the Modified Mini-Mental State Examination. The standardized protocol used for cognitive assessments in the HAAS has been described previously [17,20]. For this study, two manipulations were performed to prepare the CASI data for multistate transition modeling, as has been done previously [14,15]. Individual CASI scores were reverse scored so that lower scores represent better cognition (for example, a score of 100 on the CASI = 0 CASI “errors”). Secondly, CASI error scores were grouped by intervals of three (for example, 0–2 errors, 3–5 errors, and so forth) forming a series of cognitive states ranging from high cognition/low errors to impaired cognition/high errors. Each grouping is considered as a global cognitive state where transition models can be used to estimate the probabilities of transitioning, including improvement, stability, and decline, from baseline states to consequent cognitive states at follow-up assessments.

### Frailty index

A frailty index (FI), based on the accumulation of deficits, was created using a standard procedure [21]. Variables were selected as health deficits if they were associated with health status, accumulated with age, and had a prevalence in the sample greater than 1% but less than 80%. Each variable was dichotomized (0 for absent; 1 for present). These deficits were then summed and here divided by 48, that being the total number of deficits considered (see Appendix A in Additional file 1). Thus, a participant with no missing measurements and 24 deficits would be given a FI score of 24/48 = 0.50. We treated the FI as missing for participants in whom data were missing on more than 20% of the items.

### Social vulnerability index

Using the procedure outlined to create a FI [21], 18 social variables were selected from the baseline wave to construct a measure of overall social vulnerability (Social Vulnerability Index; SVI). This approach has been demonstrated to predict a range of health outcomes, including cognition and survival [22,23]. All HAAS variables that could be considered as social deficits were included in a dichotomized format (0 for absent; 1 for deficit) including living alone, social networks, and marital status (see Appendix B in Additional file 1). As with the FI, the social deficits were summed and divided by the total number of health deficits considered, producing a score between 0 and 1.

### Protective factors index

To evaluate the cumulative impact of a number of protective factors, a protection index (PI) was generated using baseline variables that were considered to be related to positive health outcomes. A previous study using this approach demonstrated that the aggregate effect of protective factors can influence age-related health outcomes [24]. Within the HAAS, 20 baseline variables that might offer protection to age-related cognitive decline were selected including physical exercise [25,26], the use of antihypertensive medications [27], availability of health services, not smoking cigarettes [28], moderate alcohol consumption [29], self-rated good health [30], healthy body weight [31], and others (see Appendix C in Additional file 1). As with the FI and SVI, an accumulation approach was used [21], in which selected items were dichotomized, summed, and divided by the total number of items considered. Note that in this index, higher scores are positive, reflecting that more protective factors are present (whereas in the other two indices higher scores are negative).

### Standard protocol approvals, registrations, and patients consents

Data collection was approved by the Kuakini Medical Center Institutional Review Board, with informed consent provided by all participants. Approval for the secondary analyses came from the Research Ethics Committee of the Capital District Health Authority, Halifax, Nova Scotia, Canada.

### Statistical analysis

The primary outcomes of the analyses were transitions in cognitive status and mortality at the first and second follow-up waves. Analyses take into consideration the baseline state, and then estimate the probability of transitions (staying at the same score, as well as degrees of worsening and improvement) from each of the states at baseline to the follow-up cognitive states using a Poisson approximation [14,15]. The probability of death was simultaneously modeled using a logistic function [14,15]. The incorporation of death into the transition models is essential, as longitudinal studies with older subjects are inherently predisposed to attrition via death [32], and failing to incorporate death in analyses can lead to biased estimates [33].

**Figure 1:**
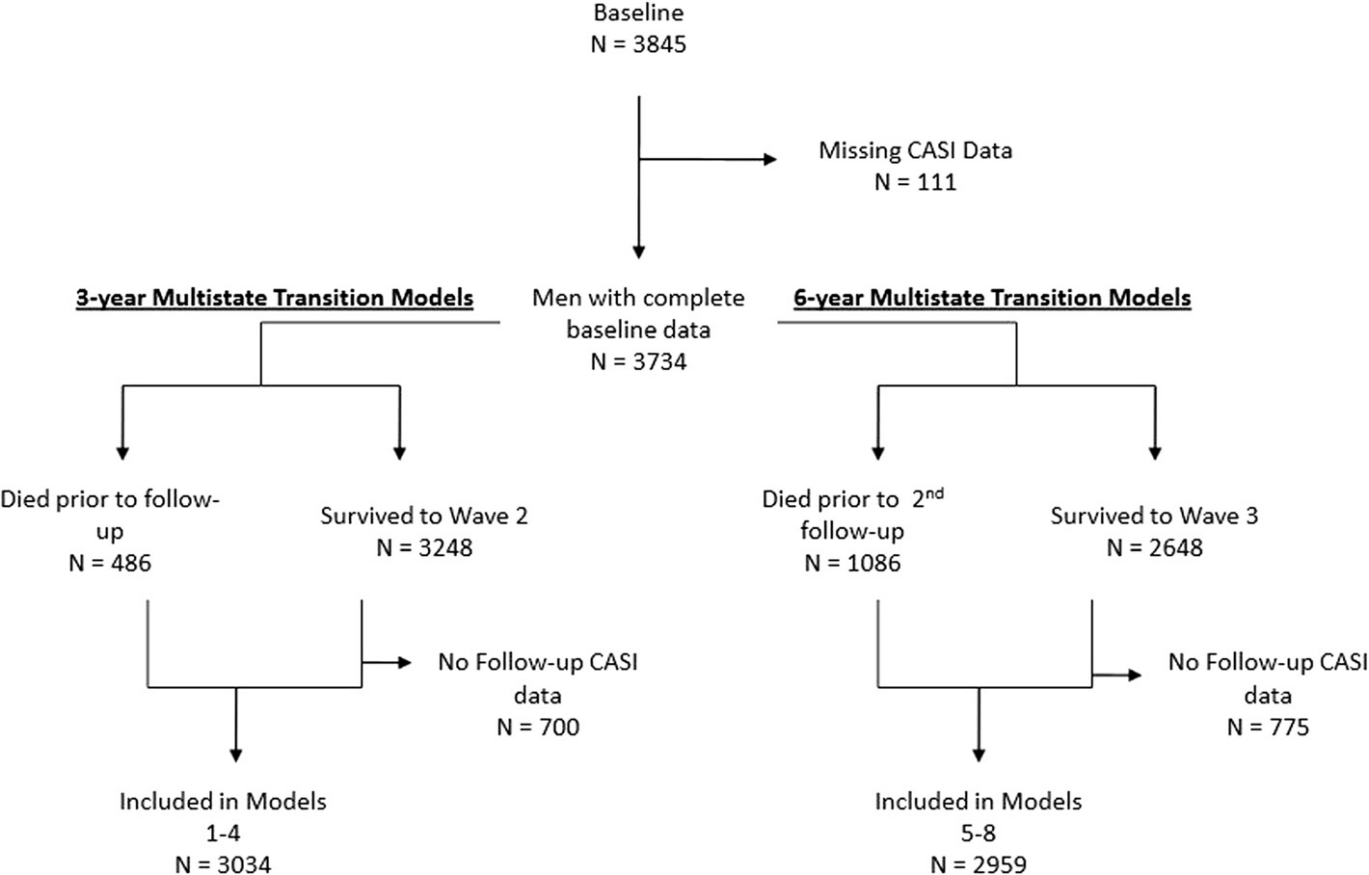
**Study flowchart for two sets of models: 3-year transition models on the left; 6-year transition models on the right.** CASI, Cognitive Abilities Screening Instrument.

For this study, the effects of the baseline level of frailty, social vulnerability, and protective factors were evaluated for two time intervals (3 and 6 years), controlling for age (in years, measured as a continuous variable, centered on the grand mean of 78 years) and education (measured in years, continuous, centered on the grand mean of 10.5 years). To aid in interpretation of betas (β) and odds ratios, each of the index variables were transformed by multiplying by 10 so that results relate to 10% increases in the index variables. Figure 1 contains the participant flow chart for the two series of models including number of decedents within each interval. For each interval, index variables were modeled separately to analyze the effects of each of the indices on the outcomes, independent of the other indices. Models included all covariates (age, education, frailty, social vulnerability, protective factors). The outputs of the multivariable Poisson regression models are parameters (β) that can be used to analyze the effects that covariates have on the transition from any baseline state to any follow-up cognitive state or death. A positive coefficient indicates that increases in the predictor variable are on average related to increases in CASI errors. When the β coefficients are negative, the variable can be considered to have, on average, a protective effect of changes in cognition. As with standard regression analyses, the β values represent the expected impact on the outcome variable based upon a one-step increase in the predictor variable. To ease interpretation, all coefficients from the logistic regression models were transformed to odds ratios; Akaike information criterion and the Bayesian information criteria were calculated for each Poisson model as indicators for relative quality of the model fit (lower scores indicate better model fit). Similarly, −2_log_ likelihood was used for logistic regression models. All analyses were performed in SPSS 18.0 (SPSS Inc., Chicago).

## Results

At baseline, the average age was 77.9 years (standard deviation (SD) = 4.7; range 71 to 93; median = 76). The average number of years of education was 10.5 (SD = 3.2; range 1 to 24; median = 11). Those who survived across each of the two intervals were younger and had slightly more education (Table 1). The average FI and the average SVI scores at baseline were lower for survivors across the two time intervals, and the average PI was higher (Table 1). In the first interval from baseline to wave 2, the average time between assessments was 2.9 years (SD = 0.3). Surviving men who did not have follow-up cognitive testing were excluded (n = 700) and 498 men had died. The three single-index models indicated that each of the covariates had a significant effect on cognitive transitions (Table 2), The FI and the SVI were each associated with cognitive decline (FI: β = 0.18, 95% confidence interval (CI), 0.08 to 0.29; SVI: β = 0.16, 95% CI, 0.08 to 0.23), whereas the protective factors were associated with higher probability of cognitive improvement (PI: β = −0.16, 95% CI, −0.24 to −0.09). For mortality within the first time interval, the models indicated that education was not significantly associated with death (*P* > 0.05). When all of the covariates were entered into a multi-index model, each continued to have an impact on cognitive transitions, although education and social vulnerability were not significantly associated with death (Table 3).

**Table 1:** **Data characterization at baseline for the full cohort and stratification by mortality status at follow-ups (3 and 6 years)**

	**n**	**Age (years)**	**Education (years)**	**Frailty index**	**Social vulnerability index**	**Protection index**	**CASI error at baseline**
Full cohort	3,845	77.9 (4.7)	10.5 (3.2)	0.15 (0.09)	0.25 (0.13)	0.36 (0.12)	17.8 (16.4)
Alive at first follow-up	3,347	77.4 (4.4)	10.6 (3.2)	0.14 (0.08)	0.25 (0.13)	0.37 (0.11)	15.5 (12.5)
Dead at first follow-up	498	80.9 (5.4)	9.8 (3.3)	0.19 (0.10)	0.30 (0.15)	0.30 (0.14)	33.1 (27.7)
Alive at second follow-up	2,759	76.9 (4.0)	10.7 (3.2)	0.14 (0.08)	0.24 (0.12)	0.37 (0.11)	14.1 (9.9)
Dead at second follow-up	1,086	80.3 (5.3)	10.0 (3.2)	0.18 (0.10)	0.29 (0.0.13)	0.32 (0.13)	27.4 (24.1)

Values are shown as mean (standard deviations). CASI, Cognitive Abilities Screening Instrument.

**Table 2:** **Parameter estimates for Poisson models examining cognitive transitions over 3 and 6 years**

	**Model 1 Frailty index**	**Model 2Social vulnerability index**	**Model 3 Protective factors index**	**Model 4 All indices**
**Wave 1 to wave 2**				
Intercept	1.93 (1.72, 2.14)	1.80 (1.58, 2.02)	2.81 (2.47, 3.15)	2.13 (1.72, 2.55)
Baseline cognitive state	0.90 (0.87, 0.94)	0.91(0.88, 0.95)	0.90 (0.87, 0.94)	0.88 (0.84, 0.92)
Age	0.097 (0.074, 0.12)	0.092 (0.068, 0.12)	0.10 (0.08, 0.13)	0.089 (0.065, 0.11)
Years of education	−0.096 (−0.12, −0.07)	−0.084 (−0.11, −0.057)	−0.083 (−1.10, −0.055)	−0.085 (−0.11, −0.057)
Frailty	0.184 (0.08, 0.29)			0.15 (0.052, 0.26)
Social vulnerability		0.16 (0.088, 0.23)		0.13 (0.061, 0.21)
Protective factors			−0.16 (−0.24, −0.087)	−0.11 (−0.19, −0.036)
Model fit statistics				
BIC	11,776	12,183	11,834	11,674
AIC	11,747	12,213	11,863	11,633
**Wave 1 to wave 3**				
Intercept	2.40 (2.12, 2.68)	2.60 (2.31, 2.88)	3.77 (3.33, 4.22)	2.95 (2.42, 3.48)
Baseline cognitive state	1.14 (1.08, 1.19)	1.14 (1.08, 1.19)	1.13 (1.08, 1.19)	1.12 (1.07, 1.18)
Age	0.19 (0.16, 0.23)	0.19 (0.16, 0.22)	0.20 (0.16, 0.23)	0.19 (0.16, 0.22)
Years of education	−0.075 (−0.110, –0.039)	−0.062 (−0.097, −0.027)	−0.057 (−0.09, −0.021)	−0.057 (−0.09, −0.021)
Frailty	0.40 (0.27, 0.54)			0.38 (0.24, 0.51)
Social vulnerability		0.14 (0.051, 0.24)		0.10 (0.01, 0.20)
Protective factors			−0.21 (−0.31, −0.11)	−0.19 (−0.28, −0.087)
Model fit statistics				
BIC	10,560	10,881	10,620	10,534
AIC	10,533	10,853	10,593	10,496

Values are shown as β (95% confidence interval) apart from AIC (Akaike information criterion) and Bayesian information criteria (BIC).

**Table 3:** **Logistic regression models examining odds of death over 3 and 6 years**

	**Model 1 frailty index**	**Model 2 social vulnerability index**	**Model 3 protective factors index**	**Model 4 all indices**
**Wave 1 to wave 2**				
Baseline cognitive state	1.09 (1.05,1.12)	1.10 (1.07, 1.14)	1.08 (1.05, 1.11)	1.06 (1.03, 1.10)
Age	1.09 (1.06,1.12)	1.08 (1.06, 1.11)	1.09 (1.06, 1.12)	1.08 (1.05, 1.12)
Years of education	1.02 (0.975, 1.06)*	1.04 (1.00, 1.08)*	1.03 (0.99, 1.08)*	1.03 (0.99, 1.08)*
Frailty	1.71 (1.51, 1.95)			1.71 (1.50, 1.95)
Social vulnerability		1.15 (1.06, 1.26)		1.06 (0.97, 1.17)*
Protective factors			0.77 (0.69, 0.86)	0.77 (0.68, 0.87)
Model fit statistics				
−2_log_ likelihood	1,771.41	2,000.19	1,876.42	1,732.20
**Wave 1 to wave 3**				
Baseline cognitive state	1.09 (1.06, 1.13)	1.11 (1.08, 1.14)	1.10 (1.07, 1.12)	1.08 (1.04, 1.11)
Age	1.12 (1.09, 1.14)	1.11 (1.09, 1.13)	1.12 (1.09, 1.14)	1.11 (1.09, 1.13)
Years of education	1.02 (0.99, 1.05)*	1.04 (1.01, 1.07)	1.05 (1.02, 1.08)	1.04 (1.00, 1.07)*
Frailty	1.73 (1.57, 1.92)			1.71 (1.54, 1.89)
Social vulnerability		1.21 (1.13, 1.29)		1.13 (1.05, 1.21)
Protective Factors			0.79 (0.73, 0.86)	0.81 (0.74, 0.89)
Model fit statistics				
−2_log_ likelihood	3,031.24	3,333.43	3,168.75	2,980.07

Values are shown as odds ratios (95% confidence intervals) except for –2_log_ likelihood. *Not significant at *P* = 0.05 level.

The second iteration of models examined the cognitive transition over a longer interval, from baseline to wave 3; the average time elapsed was 6.1 years (SD = 0.6). During this interval, 1,086 of the men had died. Individuals who neither died within 6 years nor had follow-up cognitive testing were excluded (n = 775). The single index multivariate Poisson models for this interval produced results similar to the 3-year interval, with significant effects for each of the indices on both cognitive transitions and death. The multi-index model indicated that education was not significantly associated with death after accounting for the covariates (Table 3).

The β coefficients from the Poisson regression models can be used to predict future CASI error states (fCASI):

$$ \mathrm{fCASI} = \mathrm{intercept} + \upbeta \left(\mathrm{baseline}\ \mathrm{cognitive}\ \mathrm{state}\right)+\upbeta \left(\mathrm{education}\right)+\upbeta \left(\mathrm{age}\right)+\upbeta \left(\mathrm{F}\mathrm{I}\right)+\upbeta \left(\mathrm{S}\mathrm{V}\mathrm{I}\right)+\upbeta \left(\mathrm{PI}\right) $$

For example, given the results of multi-index model for the 3-year interval, an average male (78 years old with 10.5 years of education) in CASI error group 7 at baseline, a frailty index of 0.4, social vulnerability index of 0.3, and a protective index of 0.2 would have the predicted transition in the following 3 years:

$$ \begin{array}{c}\mathrm{fCASI} = \mathrm{intercept}+\upbeta \left(\mathrm{baseline}\ \mathrm{cognitive}\ \mathrm{state}\right)+\upbeta \left(\mathrm{education}\right)+\upbeta \left(\mathrm{age}\right)+\upbeta \left(\mathrm{F}\mathrm{I}\right)+\upbeta \left(\mathrm{S}\mathrm{V}\mathrm{I}\right)+\upbeta \left(\mathrm{PI}\right)\\ {}=2.13+0.88\ (7)+-0.085\ (0)+0.089\ (0)+0.15(4)+0.13\ (5)+\left(-0.11\right)\ (2)\\ {}=9.32\end{array} $$

The zero scores for age and education are due to the fact that these variables were centered at the cohort’s average. For the index variables, scores are multiplied by 10 so that β represents a 10% increase in the index scores. The model would estimate that the individual with those characteristics would move from a CASI error group of 7 (considered cognitively intact) to an error group of 9, which would be associated with a CASI score of 73. In this worked example, the model would predict incident cognitive impairment as prior HAAS analyses determined a CASI score <74 as an accurate cut-point with ~80% sensitivity and 90% specificity for identifying dementia [34].

## Discussion

In this study, we examined cognitive changes in older Japanese-American men in the HAAS in relation to frailty and social vulnerability, and modeled the impact of protective factors on near-term (3-year) and medium-term (6-year) outcomes. Worse health status (represented by higher FI scores) and greater social vulnerability (higher SVI scores) were associated with a greater risk of death, and a greater chance of cognitive decline at 3 years. These effects, however, were partially mitigated by a range of health protective behaviors, even adjusting for education. At 6 years, the results were similar in significance and direction (FI and SVI increased CASI errors, the PI decreased errors), but were stronger. This study illustrates that the many factors that influence cognition can be handled in a tractable model, and illustrates the importance of considering general health status (frailty), social vulnerability, and protective factors when examining changes in cognition in older adults.

This approach has implications for modeling the epidemiology of late-life cognitive decline. Particularly at a time when new risk factors are appearing frequently [35-37], having a means of understanding the overall context of how risk operates in late-life disorders is important. Just as it is a tenant of everyday geriatric practice that disease presents differently in the face of frailty, so might we expect that estimating disease risk must also take into account the impact of multiple, interacting medical and social problems when they are present, as they commonly are in late life. As we develop a better understanding of changes that occur across the lifespan and the shared causal mechanisms in age-related diseases, health researchers should rely less on traditional statistical approaches that were designed with assumptions of linearity and independence. As there is a growing appreciation of a systems biology approach within medicine and the health sciences [38-41], methods from other scientific disciplines (for example, physics, network science, and computer science) should be considered to address these complexities.

PI is a relatively new approach to understanding how the accumulation of extrinsic factors can impact health outcomes [24]. Here, the PI was consistently associated with positive cognitive outcomes across both time intervals, even when accounting for baseline cognition, age, education, social vulnerability, and frailty. A recent publication with a PI found that the accumulation of protective factors had a positive impact of health transitions and mortality outcomes [24], but did not investigate the impact on cognition. Our findings provide evidence that the more protective factors that an individual possesses in late life, regardless of precisely which elements are accrued, the greater the protective effects on cognition. While research that focuses on individual protective factors is undoubtedly important, it is also necessary to develop understanding of how these factors work in combination with each other to protect against cognitive decline in old age.

Frailty was a significant predictor of cognitive states at 3 and 6 years from baseline. Recent studies have found similar findings in other cohort studies using the FI [14,15] as well as the Fried phenotypic approach to measuring frailty [41-46]. The findings of the current study extend this body of literature by examining cognitive change and frailty across multiple time points in a cohort while controlling for important confounders and accounting for attrition due to mortality. At present, even though the mechanisms underlying the relationship between frailty and cognitive decline are not clear, some clues about brain health are emerging. For example, Buchman and colleagues found that the rate of progression of frailty was significantly associated with the presence of macroinfarcts, Alzheimer's disease and Lewy body pathology, and nigral neuronal loss [47]. When considered together in a single model, the neuropathology in the study explained 8% of the variance in the rate of progression of frailty, after accounting for demographic variables.

The results of the transition models also illustrated the cumulative effect of social factors on both cognitive decline and mortality. When considered in relation to age and education, the accumulation of social deficits was significantly associated with cognitive decline at wave 2 and death by wave 3. The results coincide with recent research that indicated the importance of examining multiple social factors in parallel. Shankar and colleagues found significant interaction effects of isolation and loneliness, indicating the need to study the effects of these social factors simultaneously [48]. Here, we have compiled a series of social factors and found that an accumulation of social deficits is predictive of future cognitive transitions, warranting further research attention to potential interventions that may lessen the burden of social vulnerability in older adults. While feelings of loneliness have been shown to predict the onset of dementia in community-living older persons [49], more work is required to further understand how external factors can accumulate and synergistically impact health outcomes.

For transitions in cognition across both intervals, on average, education had protective effect. This reflects the well-known relationship between education and cognitive status in late life, in which more education is associated with better cognitive outcomes. However, results with respect to the impact of education on the trajectory of age-related cognitive decline have been mixed [50]. The results of this study add to this debate, and illustrate that, on average, increases in education are associated with better cognitive outcomes over 3 and 6 years, even after accounting for frailty, social vulnerability, and the accumulation of protective factors. Due to the complex relationships between education, health states, and global cognition in older adults, more research is needed to disentangle their relationships to develop a more robust understanding of how they interact to impact well-being. From the logistic models, education was not associated with risk of death after accounting for the other covariates. This lack of an effect is likely due to stronger influences of age, baseline cognitive state, and the index variables on mortality.

Our results must be interpreted with caution. Even though the HAAS death data are nearly complete, missing data including men too ill to participate may have impacted the estimates of the models. Second, the study used self-report data, which can be less accurate than data retrieved clinically or through laboratory tests and can be influenced by the cognitive state of the subjects. The FI was constructed using 48 items that were not strongly correlated with cognitive decline. Despite this careful approach, the FI still contained some variables that have previously been associated with risk of dementia or cognitive decline, so their combined effect on worsening cognition is not surprising. What this adds is an estimate of their combined effect, showing that risks for cognitive decline in late life need to take into account the overall degree of health [51]. Further, previous research has shown that frailty, measured in different ways, including a set of non-traditional risk factors for dementia [52,53], can be associated with future cognitive states. Even so, that work has been criticized for not being closely enough tied to causal mechanisms, thereby limiting applicability in understanding how particular interventions might work. The current report addresses this in two ways: first, it shows the type of factors, in the PI, that can mitigate risk. Second, it illustrates how the deficit accumulation approach can be employed to take into account a broader range of risk factors than often gets considered in single risk factor models. For example, if new risk factor X or novel protective factor Y were to be proposed, the importance of their impact would be enhanced if it withstood adjustment for the types of index variables proposed here.

Both the SVI and the PI might have benefitted had additional factors that were not measured in the baseline wave of the HAAS been included. Nevertheless, the effects of both these indices were significantly associated with the health outcomes at multiple time points. The optimization of these indices warrants further investigation and is of interest to our research group. By conducting transformations on the original CASI scores, cognitive states were arbitrarily defined using groupings of three. This type of transformation is computationally convenient and is unlikely to mix clinically separable states. Even so, to ensure that choice of grouping did not influence the results, models were also developed using alternative groupings (2, 4, 5); these led to similar results. Finally, these analyses only evaluated the impact of baseline measurements with future health outcomes. It is important to realize that these baseline factors also operate in a dynamic way and will also change considerably over the years. Future analyses examining cognitive changes in late life should also aim to consider factors such as frailty and social vulnerability as time-varying covariates. This type of approach would greatly assist in developing our understanding of the complex dynamics in late life.

## Conclusions

In the HAAS cohort, as in others, average cognitive function declines over time. When taking a traditional statistical approach, improvement and stability can get lost in this average decline. In contrast, the transition modeling approach that we have employed not only accounts for cognitive dynamics, but also may allow some insights into the phenomenon of late-life cognitive decline that is certainly becoming a major issue for public health. Coupled with the use of index variables, it appears to allow a critical perspective in adjudicating claims about individual risk factors. As a recent *Nature* commentary pointed out, it is essential that we employ approaches that take into account the heterogeneity of disease risk and expression in evaluating age-associated illness [54]. These considerations are motivating additional inquiries by our group.

## Additional file

Additional file 1:
**Contains appendices A-C which list the items from the Honolulu-Asia Aging Study that were used to create index variables.**

## Abbreviations

CASI: Cognitive Abilities Screening Instrument
CI: confidence interval
FI: frailty index
HAAS: Honolulu-Asia Aging Study
PI: protection index
SD: standard deviation
SVI: Social Vulnerability Index
