# Supplementary material for: Cumulative impact of health deficits, social vulnerabilities, and protective factors on cognitive dynamics in late life: a multistate modeling approach
Source: Alzheimers Res Ther. 2015 Jun 5;7(1):38. doi: 10.1186/s13195-015-0120-7 (PMC4457088; doi:10.1186/s13195-015-0120-7)
Supplement: Additional file 1: — Contains appendices A-C which list the items from the Honolulu-Asia Aging Study that were used to create index variables. [file 13195_2015_120_MOESM1_ESM.pdf]

Appendix A. Frailty index items coded 0 for absent, 1 for present.

Functional Deficits	Deficits of Diseases and Conditions	
• Difficulty walking 1/2 mile	• Previous heart attack	• Awakened at night by coughing
• Difficulty walking up 10 steps	• Angina	• Swelling of your feet or ankles
• Unable to do heavy work	• High blood pressure	• Lack of appetite
• Difficulty lifting	• Diabetes	• Feelings of fear
• Difficulty reaching out	• Heart disease	• Restless sleep
• Difficulty gripping with hands	• Cholecystitis or gall stones	• Myocardial infarction
• Hearing impairment	• Polyps of large bowel	• Peripheral vascular disease
• Vision impairment	• Stomach or peptic ulcer	• Pulmonary disease
• Dizziness	• Prostate problems	• Fatigue past 2 weeks
• Unusually irritable	• Kidney disease	• Regular cough
• Trouble with focus	• Cancer (except skin)	• Bronchitis (acute or chronic)
• Feelings of exhaustion	• Arthritis	• Bone Fracture
• Lack of hope for future	• Cataract	• Shingles
• Feelings of loneliness	• Signs of parkinsonism	• Polypharmacy (>5 medications)
• Trouble with initiation	• Angina pectoris	• Dissatisfied with quality of life

Appendix B. Social Vulnerability index items coded 0 for absent, 1 for present.

Social Deficits		
• When lonely, there is no one to talk to	• Do not have somebody to talk to about important decisions	• No close friends
• I rarely meet/talk with family or friends	• People do not talk to you about important decisions	• Do not participate in any groups
• Cannot find somebody help w/ daily chore	• Don't have people to help with things like shopping, etc.	• Hearing cause difficulty when visiting friends
• Do not know somebody that can turn to with personal problem	• Living alone	• Do not do regular volunteer work
• Do not trust at least one person's advice	• See few many relatives once a month	• Marital status - Single
• Could not find someone to care for house	• Feel close to few people or relatives	• Low yearly household income (<15000)

Appendix C. Protection index items coded 0 for absent, 1 for present.

Protective Factors		
• Healthy Body mass index<br>• (BMI = 18.5-25)	• Availability of doctor	• Moderate Alcohol Intake per month (oz)
• Calcium-channel blockers Use	• Availability of hospital care	• Good self-rated general health
• Take vitamins/supplements	• Regular exercise	• Able to tolerate job stress
• Recently visited a dentist	• Non-smoker	• Regular aspirin consumption
• Ace-inhibitors Use	• Physical Active	• Speak Japanese often
• Beta-blockers Use	• Satisfied with quality of life	• Read or write Japanese now
• High yearly income (>$30000)	• Climbed more than 6 flights of stairs per day	
